# Establishing CD19 B-cell reference control materials for comparable and quantitative cytometric expression analysis

**DOI:** 10.1371/journal.pone.0248118

**Published:** 2021-03-19

**Authors:** Lili Wang, Rukmini Bhardwaj, Howard Mostowski, Paul N. Patrone, Anthony J. Kearsley, Jessica Watson, Liang Lim, Jothir Pichaandi, Olga Ornatsky, Daniel Majonis, Steven R. Bauer, Heba A. Degheidy

**Affiliations:** 1 Biosystems and Biomaterials Division, National Institute of Standards and Technology (NIST), Gaithersburg, Maryland, United States of America; 2 Office of Tissues and Advanced Therapies, Center for Biologics Evaluation and Research, Food and Drug Administration (FDA), Silver Spring, Maryland, United States of America; 3 Applied and Computational Mathematics Division, NIST, Gaithersburg, Maryland, United States of America; 4 Fluidigm Canada, Markham, Canada; Centre National de la Recherche Scientifique, FRANCE

## Abstract

In the field of cell-based therapeutics, there is a great need for high-quality, robust, and validated measurements for cell characterization. Flow cytometry has emerged as a critically important platform due to its high-throughput capability and its ability to simultaneously measure multiple parameters in the same sample. However, to assure the confidence in measurement, well characterized biological reference materials are needed for standardizing clinical assays and harmonizing flow cytometric results between laboratories. To date, the lack of adequate reference materials, and the complexity of the cytometer instrumentation have resulted in few standards. This study was designed to evaluate CD19 expression in three potential biological cell reference materials and provide a preliminary assessment of their suitability to support future development of CD19 reference standards. Three commercially available human peripheral blood mononuclear cells (PBMCs) obtained from three different manufacturers were tested. Variables that could potentially contribute to the differences in the CD19 expression, such as PBMCs manufacturing process, number of healthy donors used in manufacturing each PBMC lot, antibody reagent, operators, and experimental days were included in our evaluation. CD19 antibodies bound per cell (ABC) values were measured using two flow cytometry-based quantification schemes with two independent calibration methods, a single point calibration using a CD4 reference cell and QuantiBrite PE bead calibration. Three lots of PBMC from three different manufacturers were obtained. Each lot of PBMC was tested on three different experimental days by three operators using three different lots of unimolar anti-CD19PE conjugates. CD19 ABC values were obtained in parallel on a selected lot of the PBMC samples using mass spectrometry (CyTOF) with two independent calibration methods, EQ4 and bead-based calibration were evaluated with CyTOF-technology. Including all studied variabilities such as PBMC lot, antibody reagent lot, and operator, the averaged mean values of CD19 ABC for the three PBMC manufacturers (A,B, and C) obtained by flow cytometry were found to be: 7953 with a %CV of 9.0 for PBMC-A, 10535 with a %CV of 7.8 for PBMC-B, and 12384 with a %CV of 16 for PBMC-C. These CD19 ABC values agree closely with the findings using CyTOF. The averaged mean values of CD19 ABC for the tested PBMCs is 9295 using flow cytometry-based method and 9699 using CyTOF. The relative contributions from various sources of uncertainty in CD19 ABC values were quantified for the flow cytometry-based measurement scheme. This uncertainty analysis suggests that the number of antigens or ligand binding sites per cell in each PBMC preparation is the largest source of variability. On the other hand, the calibration method does not add significant uncertainty to the expression estimates. Our preliminary assessment showed the suitability of the tested materials to serve as PBMC-based CD19+ reference control materials for use in quantifying relevant B cell markers in B cell lymphoproliferative disorders and immunotherapy. However, users should consider the variabilities resulting from different lots of PBMC and antibody reagent when utilizing cell-based reference materials for quantification purposes and perform bridging studies to ensure harmonization between the results before switching to a new lot.

## Introduction

Cell-based therapies have emerged as potential novel approaches to treat many diseases and regenerate damaged tissues [1–3]. At present, immunotherapy has been widely explored and shows promising results in treating cancer patients [4–7]. One immunotherapy approach uses an engineered version of each patients T cells modified to attack cancer cells, these modified cells are known as Chimeric Antigen Receptor (CAR)-T cells. Several clinical trials have demonstrated activity of CAR-T cells against multiple subtypes of B-cell lymphoma [8–11], including follicular Lymphoma [12,13], diffuse large B-cell lymphoma (DLBCL) [12,14], chronic lymphocytic leukemia [7], and refractory acute B- cell lymphoblastic leukemia [15–18]. Within this greater context, flow cytometry plays an increasingly important role in disease diagnosis, and monitor patients after treatment for assessing the efficacy of the given therapy [19–24]; and providing guidance for selecting the appropriate targeted therapies in B-Lymphoblastic Leukemia [17,18]. Furthermore, recent studies have highlighted the crucial role of flow cytometry in identifying patients who will benefit the most from the immunotherapeutic products [25,26]. This is mainly due to the ability of flow cytometry to quantify the expression level of important markers on malignant cells. Currently, multiple new emerging treatment options for B- cell malignancies rely on evaluating CD19 expression either to initiate the process [11,14,27] or select patients who will benefit from targeted monoclonal antibody therapies [28,29]. Pillai et al., have published data demonstrating that anti-CD19 CAR T-cell therapy is effective for CD19-dim B-lymphoblastic leukemia compared with CD19-normal or -bright expression [25]. Ramakrishna and co-workers have reported that low CD22 expression negatively impacts in vitro and in vivo anti-CD22 CART functionality and impairs in vivo CART persistence [26]. There is an urgent need to develop a robust method to better quantify the expression level of crucial markers that are directly involved in crucial processes such as patient selection and critical treatment decisions in B cell malignancy [25,26,30–32]. It is equally important to characterize response of B-cell malignancies to personalized T cell immunotherapy products to ensure their safety and efficacy [16,20,21]. However, the lack of adequate biological and non-biological reference materials, as well as the complexity of the cytometer instrumentation, have resulted in few standards to improve confidence in such measurements.

The ultimate goal of comparable and quantitative flow cytometry is to determine the number of antigens or ligand binding sites associated with a cell by measuring the number of antibodies bound per cell (ABC) [23,31,33,34]. A robust flow cytometric method should therefore include proper controls and standards, i.e. particles and biological reference materials for instrument calibration, performance characterization, and standardization [35,36]. A common calibration approach uses QuantiBrite PE beads with unimolar monoclonal antibody PE conjugates to enable measurement of antigen expressions in terms of ABC value [37,38]. However, as this scheme relies on PE calibration beads, it is limited to calibrating only those channels associated with the PE fluorescence. This motivates us to consider a second calibration approach that uses a biological reference cell material known to possess a fixed number of well characterized CD4 protein biomarkers [32,39–42]. Several studies showed variations in CD4 expression on normal T cells between healthy subjects [37,43] which highlight the need of a robust reference material with consistent and stable antigen expression for the purposes of quantitative flow cytometry.

Our multi-year efforts on quantifying CD4 expression levels on human T lymphocytes aimed at identifying reliable reference biomarkers. We demonstrated consistent CD4 expression on Cyto-Trol Control Cells by three independent methods: flow cytometry, mass cytometry (CyTOF), and quantitative mass spectrometry [38,42]. Hence, the lyophilized Cyto-Trol Control Cells with the three method-averaged CD4 ABC value of 40,500 make this alternative quantification approach accessible to all channels of flow cytometers [38,42].

To address the urgent need for population specific reference materials that can be used as a reference upon which other relevant markers could be quantified, this study was designed to characterize CD19 expression levels on three commercially available, lyophilized or dried-down peripheral blood mononuclear cells (PBMC). As these PBMC preparations are both widely accessible to users and expected to exhibit less variability than fresh and cryopreserved PBMCs from healthy individuals, they are candidates for the development of CD19 reference controls. Our study aims to support their future development into reference standards.

The key outputs of this work are to quantify CD19 expression, and thoroughly explore the associated variations in each PBMC preparation and assess the impact of uncertainties arising from all aspects including cell manufacturing, the instrumentation, antibody reagent, and sample preparation. Because our work focuses on characterization of PBMC preparations in support of reference material development and standardization, uncertainty quantification (UQ) plays a fundamental role in our analysis. In more formal metrology settings, this process involves an in-depth analysis of the measurement tools used to characterize the standards, material manufacturing processes, and/or considerations associated with SI traceability. However, cell-based references, e.g. for cytometry, are challenging to develop because inherent biological variability and complex cell-optical-instrument interactions make it difficult to accurately perform uncertainty analyses. A secondary objective of this study is therefore to develop mathematical tools that support more general standardization of cell-based reference materials for cytometry measurements.

## Material and methods

### Cell samples and reagents

PBMC preparations including the respective reconstitution buffers were purchased from three different manufacturers. (PBMC-A was obtained from Beckman Coulter (Fullerton, CA) as described in Wang et al., 2012 [38] Additionally, two PBMC preparations were purchased from two different manufacturers: BD Biosciences (San Jose, California) and Bio Legend (San Diego, California). Each manufacturer used their own lyophilization protocol either to freeze or to dry down peripheral blood mononuclear cells. PBMC -B was produced from a single healthy blood donor. Whereas, PBMC-A and PBMC-C were manufactured from pooled donor samples (a minimum of three normal donors). Three different lots of each PBMC preparation made by each manufacturer were used in this study. Each PBMC preparation was reconstituted in an appropriate buffer following each manufacturers’ instruction. After addition of an appropriate volume of reconstitution buffer, vials containing cell suspensions were placed on the shaker for 30 minutes to ensure homogenous mixing.

#### Flow cytometry reagent

Three different production lots of unimolar custom-made anti-CD19 PE conjugate (CD19-PE 1:1, clone HIB19, Catalog #: 663016), unimolar custom-made anti-CD4 PE conjugate (CD4-PE 1:1, clone SK3, Catalog #: 660503) and a single lot of QuantiBrite PE Quantitation kits (Catalog #: 340495) were purchased from BD Biosciences.

#### CyTOF reagent

All antibodies were obtained from Fluidigm Maxpar Human Peripheral Blood Basic I Phenotyping Panel Kit (Catalog #: 201302). For cell staining protocols, Cell Staining Buffer (CSB) (Catalog #: 201068), Intercalator-Ir (Catalog #: 201192A, 125 μM), water (Catalog #: 201069), and Cell Acquisition Solution (CAS) (Catalog # 201240) were all acquired from Fluidigm (San Francisco, Ca). Pierce^™^ 16% formaldehyde (Catalog #: 28908) was purchased from Thermo Fisher Scientific (Waltham MA) to make a working concentration of 1.6% in PBS. Reagents for sample analysis on the Helios mass cytometer included Tuning Solution (Catalog #: 201072), EQ^™^ Four Element Calibration Beads (EQ4, Catalog #: 201078), and Washing Solution (Catalog #: 201070) were also acquired from Fluidigm. Five Antibody Binding Capacity (ABC) calibration beads were synthesized using dispersion polymerization, independently characterized for the number of metal atoms per bead [44] and combined for use in mass cytometry. The five beads feature a roughly equivalent amount of Ce, with zero and then logarithmically increasing amounts of La, Eu, Ho, and Lu.

### Flow cytometry antibody titration

An appropriate volume from each cell suspension as specified by the manufacturer was added to separate tubes for subsequent single staining with either CD19-PE 1:1 or CD4-PE 1:1. Titration curves for CD19-PE 1:1 and CD4-PE 1:1 were generated with different amount of antibody added to single staining tubes (5 μl, 10 μl, 15 μl, 20 μl, 25 μl and 30 μl) for each PBMC preparation. Antibody concentration was selected to ensure staining saturation (meaning small changes in antibody concentration have little effect on median fluorescence intensity (MedFI) of stained cells) and used thought out the entire study.

### Cytometer calibration and cross experiment harmonization

The flow cytometric measurements were performed using FACSCanto 10 equipped with FACS Diva software for data acquisition and analysis (BD Biosciences). Cytometer setup and tracking beads (CST, BD Biosciences) were used for daily quality control and setup of initial PMT voltages. On the first day of experiment, PMT voltage of the PE channel was optimized to ensure the MedFI of unstained cells is approximately 2.5 times above the electron noise of the PE channel according to previously published protocol [45]. QuantiBrite PE beads were then run; the resulting MedFI values of the four PE intensity beads were recorded and used as target values to ensure consistency between experiments performed at different days. If needed, the PMT voltage of PE channel was slightly adjusted to ensure that the target MedFI values were achieved for instrument performance harmonization.

### Gating strategies for surface CD 19 and CD4 expression analyses

**Fig 1:**
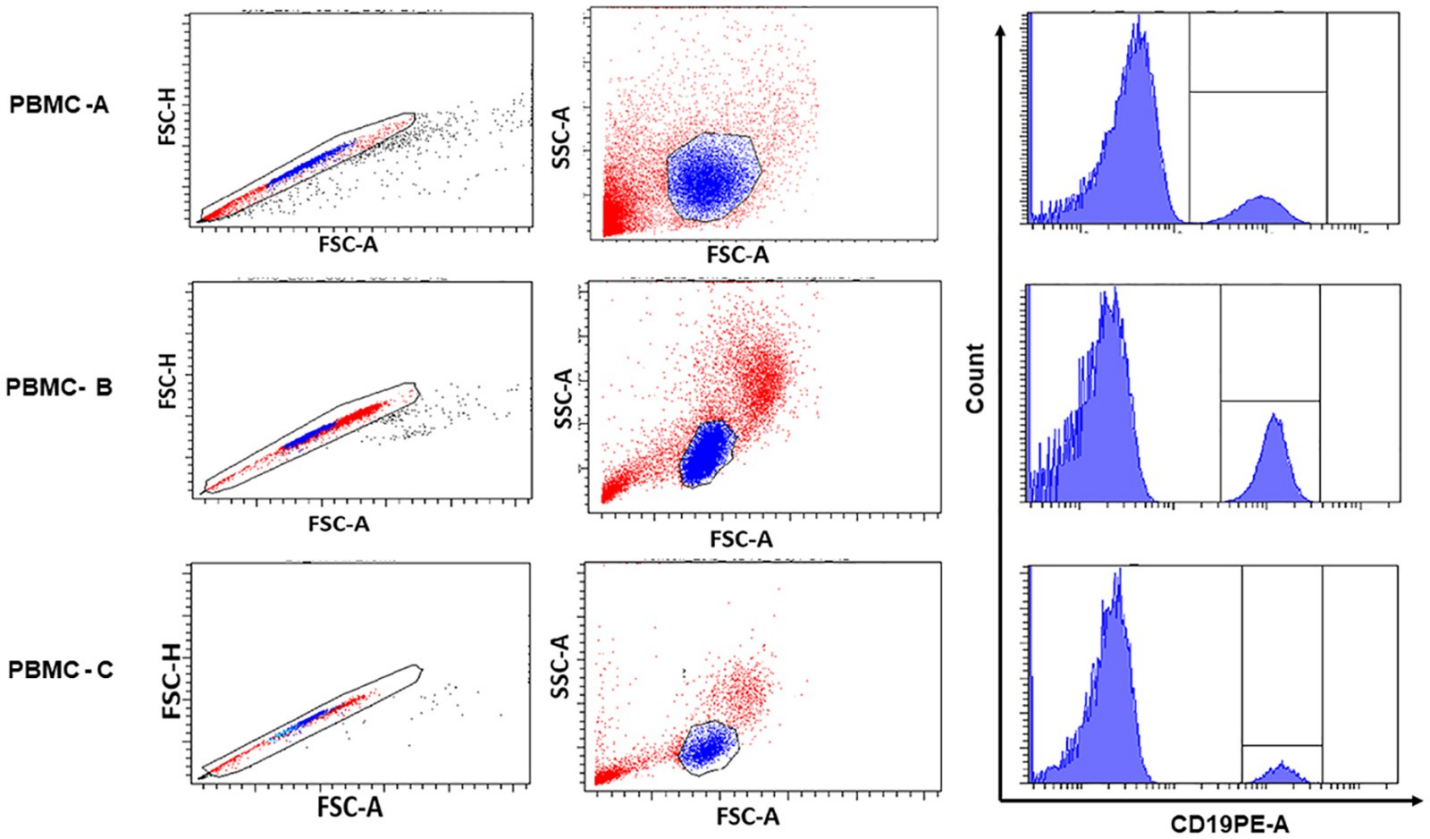
Gating Strategy to identify CD19 positive populations from PBMC-A, PBMC-B, and PBMC-C. Representative dot plots of FSC-A vs. FSC-H were used for doublets exclusion (left). The dot plots of FSC-A vs. SSC-A were then utilized to gate on lymphocyte population (middle), followed by subsequent gating on CD19 positive cells in the respective histograms (right).

To measure CD19 and CD4 expression levels, the first gate, P1, was set on singlets using a dot plot of FSC-A vs. FSC-H, and a lymphocyte gate, P2, was then defined by FSC-A versus SSC-A under the P1 gate shown in Fig 1. While gated on lymphocytes (P2), CD19 and CD4 positive cells were identified using single color histograms. CD19 histograms are shown on the right of Fig 1 as examples. The expression levels of CD4 and CD19 in terms of MedFI were collected using the proposed gating strategy (n = 18 MedFI values /per lot of PBMC (n = 3) with 2 replicates, 3 lots of antibody reagent, and 3 experimental days).

### CD19 quantification schemes using flow cytometry

CD19 ABC values were calculated using two quantification approaches. The cell-based scheme uses CD4 expression on Cyto-Trol cells (PBMC-A) as a reference biomarker with a known ABC value of 40,500, an average value from three different measurement methods previously published [38,42,45]. The CD19 ABC values were calculated using the following equation:

$${\text{ABC}}_{\text{CD}19}=({\text{MedFI}}_{\text{CD}19}/{\text{MedFI}}_{\text{CD}4})\times {\text{ABC}}_{\text{CD}4}$$ (1)

The second, bead-based quantification scheme utilizes QuantiBrite PE beads with a known number of PE molecules per bead for four bead populations [43]. Using a linear regression of Log_10_ MedFI of each bead population verse Log_10_ PE molecules per bead (y = mx + c), the MedFI value measured in the PE channel was converted to the number of PE molecules bound per cell. Because of the use of CD19 PE 1:1 for cell staining, the number of PE molecules bound per cell is equal to the number of antibodies bound per cell i.e. the CD19 ABC value.

### Sample staining and analysis using CyTOF

A pilot study was conducted on a selected subset of the PBMC samples to quantify in parallel CD19 expression using CyTOF with two independent calibration methods (EQ4 and ABC bead calibration). All samples were stained and analyzed according to the following protocol. Once three different PBMC samples were reconstituted, multiple vials of the same PBMC sample were combined into one tube; cell counting was performed followed by centrifuge with 300 xg for 5 minutes and supernatant aspiration. The pelleted cells were resuspended in the respective buffer to ensure that a 100 μL cell suspension contains 3 million cells. To this bulk suspension of cells, the master mix of antibodies from the Maxpar Human Peripheral Blood Basic I Kit excluding anti-CD19 antibody was added to a volume required for a specific number of cells being stained by the Kit. The bulk cell suspension was subsequently aliquoted into individual Eppendorf tubes as 100 μL (3 million cells) per tube. CD19 antibody was then added to each respective sample tube, and the tubes were incubated at RT for 30 minutes. CD19 antibody titration was performed ensuring CD19 quantification was carried out under saturated staining condition. Following incubation, tubes were spun down (300 xg for 5 min) and the supernatant was aspirated. Samples were washed with 1 mL CSB and spun (300 xg for 5 min), and the supernatant was aspirated. Cell samples were then fixed and intercalated overnight at 4°C by diluting the Ir-intercalator (final conc. 0.125 μM) in 1.6% formaldehyde in PBS solution (1 mL per sample). The next day, samples were spun down (800 xg for 5 min), supernatant was aspirated, and the cells were washed 2 times with CSB (2 mL, spun at 800 xg for 5 min, supernatant aspirated). The cells were counted and finally washed with PBS (same conditions as CSB wash). For sample acquisition, cell pellets were suspended in a solution of either ABC five element calibration beads or EQ four element calibration beads (diluted by 5 times) in CAS to a concentration of 0.5–1 x 10^6^ cells/mL, with 100,000 events collected. Sample collection was performed on Helios, a CyTOF system. Two CyTOF quantification approaches (EQ four calibration, and ABC bead calibration) were used to determine CD19 ABC values. Three lots of PBMC (one per company) were analyzed to determine CD19 ABC value by this method.

### Uncertainty quantification and sensitivity analysis of flow cytometric data

To better understand the various sources of uncertainty, estimate their relative contributions, and characterize typical variability of CD19 in healthy individuals, we analyzed a physically informed model of a cytometry measurement. Specifically, we postulated that for a fixed set of calibration parameters, the corresponding measurement values could be expressed as

$$v_{i,j,k,l}=m_{i}\frac{A_{j}^{2}}{1+A_{j}^{2}}+b_{k}+N_{\text{l}}(0,\epsilon^{2})$$ (2)

where *m*_*i*_ is the typical number of CD19 markers in the *i*th cell lot, $A_{j}^{2}/(1+A_{j}^{2})$ is the fraction of bound markers associated with the *j*th antibody lot, *b*_*k*_ is a bias associated with the *k*th operator, and *N*_*l*_(0, *ϵ*^2^) is the *i*th realization of a normal random variable with mean zero and variance *ϵ*^2^. The quantities *m*_*i*_, *A*_*j*_, *b*_*k*_, and *ϵ*^2^ were determined by a maximum likelihood analysis of the data separately for fixed manufacturer and calibration procedures. Given that we considered all combinations of 3 cell lots, 3 antibody lots, and 3 operators, a total of 27 measurements were available to estimate these quantities for the 6 combinations of two calibration schemes and three manufacturers.

Having determined these quantities, we next computed the sample means and sample variances

$$\mu \approx \underset{i}{\sum }m_{i}/3,\beta =\underset{k}{\sum }b_{k}/3,\;\sigma_{m}^{2}\approx 0.5\underset{i}{\sum }{\left(m_{i}-\mu \right)}^{2},\;\sigma_{b}^{2}\approx 0.5\underset{k}{\sum }{\left(b_{k}-\beta \right)}^{2}$$ (3)

and constructed synthetic noise models of the form

$$m=\mu +N(0,\sigma_{m}^{2}),\;A=U[{\text{min}}_{j}(A_{j}),{\text{max}}_{j}(A_{j})],\;b=\beta +N(0,\sigma_{b}^{2})$$ (4)

where *U*[w, w′] is a uniform random variable on the domain [w, w′], and *N*(0, σ^2^) is a normal random variable with zero mean and variance *σ*^2^. Using random number generators, we created many independent realizations of these quantities, which we inserted into Eq 2 to create a synthetic distribution measurements accounting for the aforementioned uncertainties. To further account for uncertainty in the calibration (which determines the *v*_*i*,*j*,*k*,*l*_ used in the maximum likelihood), we generated synthetic distributions of the corresponding calibration parameters and repeated the above process multiple times, combining all synthetic datasets to yield a final histogram of CD19 measurements.

A key benefit of this analysis is its ability to explicitly characterize the uncertainty due to each of the aforementioned sources, including the calibration parameters. While details are reserved for the supplemental information, we note that the quantities

$$R_{m}=\frac{{\langle {\bar{F}}^{2}\sigma_{m}^{2}\rangle }_{\phi }}{\varsigma^{2}},\;R_{F}=\frac{{\langle \mu^{2}\sigma_{F}^{2}\rangle }_{\phi }}{\varsigma^{2}},\;R_{b}=\frac{{\langle \sigma_{b}^{2}\rangle }_{\phi }}{\varsigma^{2}},\;R_{\epsilon }=\frac{{\langle \epsilon \rangle }_{\phi }}{\varsigma^{2}},\;R_{\text{mixed}}=\frac{{\langle \sigma_{m}^{2}\sigma_{F}^{2}\rangle }_{\phi }}{\varsigma^{2}}$$ (5)

characterize the relative uncertainties due to variation in the number of markers, fraction bound, bias, random errors, and mixed effects of multiple sources of uncertainty acting in concert. In these expressions, the notation 〈⋅〉_ϕ_ refers to averages with respect to the calibration parameters (which we estimate via the random sampling approach described above), $\overset{-}{F}$ and $\sigma_{F}^{2}$ refer to the mean and variance of *F*(*A*) = *A*^2^/(1+*A*^2^), and ς^2^ is the variance of a measurement accounting for all sources of uncertainty. It is also possible to show that *R*_*c*_ = 1 − *R*_*m*_ − *R*_*f*_ − *R*_*b*_ − *R*_*ϵ*_ − *R*_mixed_ ≥ 0 is the relative fraction of uncertainty due to calibration.

## Results

### Instrument harmonization for measurements performed at different days

One of the critical steps in quantitative flow cytometry measurements is to ensure that comparable and reproducible results were obtained between experimental days. To achieve this objective, a single lot of QuantiBrite PE beads was used to ensure nearly identical performance characteristic of PE channel. The MedFI values of the four PE intensity beads were utilized as target values between different days’ experiments. Our results showed that comparable MedFI values were obtained in the three experimental days with coefficient of variations (CV) < 1.0% for all intensity peaks (Low, Med-Low, Med-High and High peaks) (S1 Table). The data suggest that this harmonization approach is efficient for reducing day-to-day instrument variations and ensuring that time factor has a negligible effect on the MedFI values obtained for PBMC samples.

### Consistency of CD4 expression on Cyto-Trol control cells

The cell-based quantitative schemes utilize CD4 on T-helper cells of Cyto-Trol with a known ABC value as a reference marker for the conversion of the measured MedFI values to ABC values [38,39]. This method relies on the consistency of CD4 expression on Cyto-Trol cells. As an assurance step and prior to using CD4 expression in PBMC-A as a reference marker for CD19 ABC value determination, we tested the consistency of CD4 expression on three different lots of Cyto-Trol cells (PBMC-A) stained with three different lots of CD4 PE 1:1 over three experimental days operated by three individuals. Eighteen CD4 MedFI values were collected per lot of PBMC (2 replicates, 3 lots of antibody reagent over 3 days). The CD4 MedFI values over two sample replicates were averaged to give a total of 27 CD4 MedFI values. These values were summarized in Table 1A.

**Table 1:** CD4 MedFI values obtained using three lots of PBMC-A and three lots of antibody reagent (CD4 PE 1:1).

**PBMC Lot**	**Reagent Lot**	**Day 1**	**Day 2**	**Day 3**	
**Lot 1**	R1	53265	52140	53634	
	R2	48977	48634	50084	
	R3	46674	46759	48323	
**Lot 2**	R1	55148	54677	56294	
	R2	50706	50680	51434	
	R3	46167	46356	46954	
**Lot 3**	R1	53578	53911	54916	
	R2	48911	49611	49674	
	R3	46927	48516	47027	
**PBMC Lot**	**Reagent Lot**	**Day / Operator**			**CV Max**
Within	Within	Across			1.6
Within	Across	Within			7.4
Across	Within	Within			2.0
Within	Across	Across			7.2
Across	Within	Across			2.1
Across	Across	Within			6.3
Across	Across	Across			6.1

CD4 MedFI values obtained using three lots of PBMC-A and three lots of antibody reagent (CD4 PE 1:1) in 3 experimental days with three different operators were provided in Table 1A. Maximal %CV was calculated and shown in Table 1B for assessing uncertainty contribution from individual variable and combined variables.

Including all variables (reagent lots, days and operators, and PBMC lots), the MedFI values of CD4 range from 46167 to 56294 (Table 1A) with overall averaged MedFI of 50370, standard deviation (SD) of 3070, and an overall % CV of 6.1. Fixing different combinations of control variables and examining the respective CV’s yields a qualitative breakdown of the total measurement variability. All CVs computed for a fixed antibody reagent lot are on the order of 2.1% or less, but they climb to between 6.1% and 7.4% when the variable of the antibody lot is included (Table 1B). P values much less than 0.05 were obtained between different lots of the antibody reagents, suggesting that this variable has a significant impact on the measured fluorescence values. On the other hand, P values of no less than 0.49 were attained between different lots of PBMC and different experimental days/operators, indicating that the differences in observed CD4 MedFI values due to these two variables are insignificant (S2 Table).

While these results suggest that the CD4 expression levels are stable on the Cyto-Trol cells, the added variation due to antibody reagent lot motivated us to directly characterize the effect of using the CD4 reference material, Cyto-Trol cells on the CD19 measurements. In particular, we used the mathematical model analysis described above to isolate the relative contribution of each source of measurement variability in the raw CD4 data. Using Eqs (2)–(4), we then generated synthetic estimates of MedFI_CD4_ appearing in Eq (1). These synthetic estimates were then used to determine CD19 values, with *R*_*c*_ [see Eq (5)] yielding the relative variation due to CD4 reference marker calibration. The additional uncertainty (relative to bead-based calibration) is on the order of 5.0% or less, consistent with the CV values reported above. Moreover, the estimated variability in CD19 measurements due to other sources is independent of the calibration method to a very good approximation. These observations suggest that the CD4 reference materials provide a reasonable and independent calibration strategy within the uncertainty thresholds so described.

### CD19 MedFI & ABC values obtained for different cell preparations

**Fig 2:**
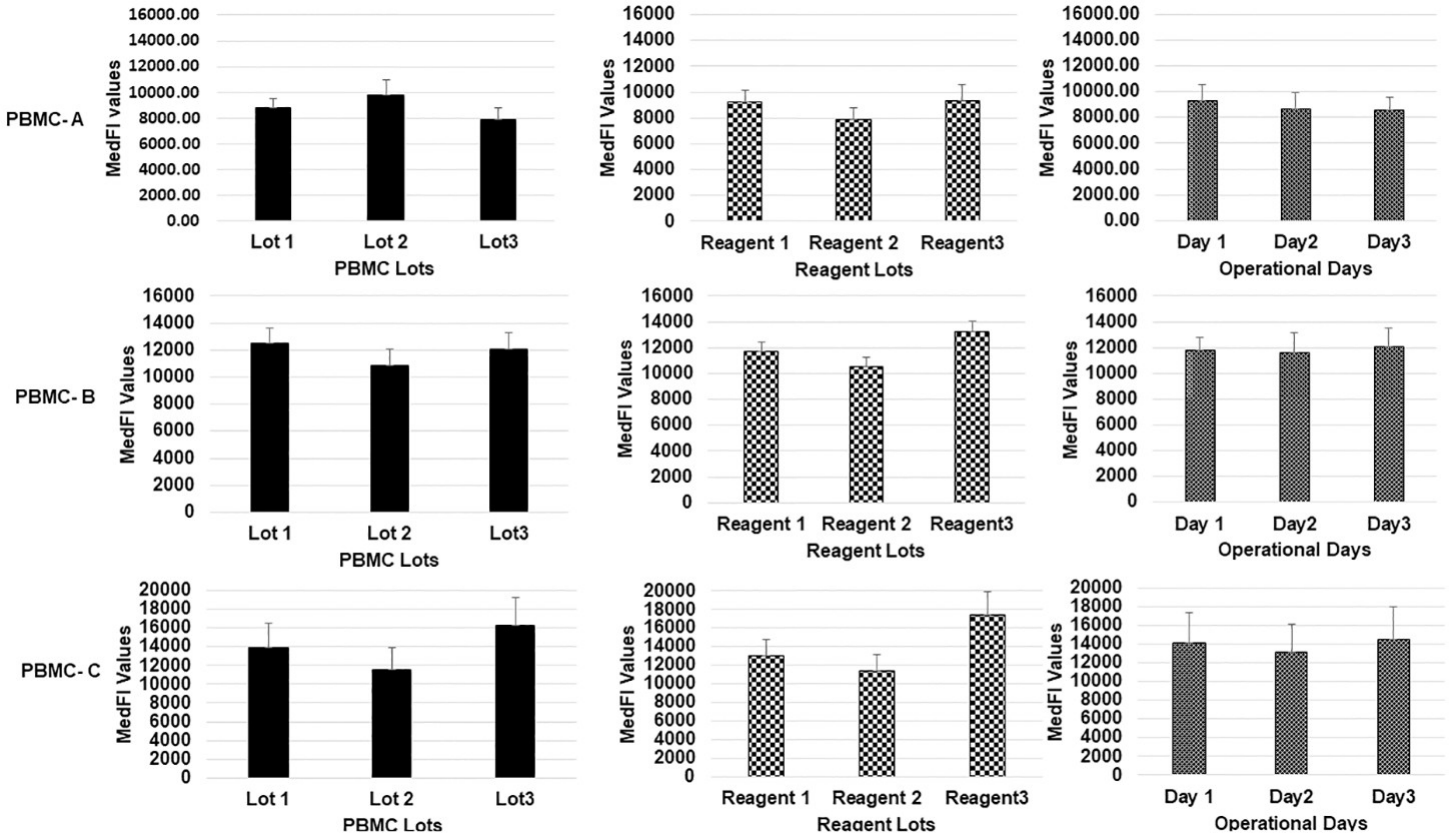
MedFI values and associated standard deviations obtained for the three PBMC preparations. MedFI values and associated standard deviations obtained for the three PBMC preparations, PBMC-A (top row), PBMC-B (middle row), and PBMC-C (bottom row). Variables contributing to variability of MedFI values include different lots of each PBMC preparation (left column), CD19 PE 1:1 antibody reagent (middle column), and experimental days with different operators (right column).

While gated on CD19+ cells as shown in Fig 1, CD19 MedFI values were recorded for each lot of PBMC (Lot 1, Lot 2 and Lot 3), using three lots of CD19 PE 1:1 reagent (R1, R2 and R3) in three different experimental days. These CD19 MedFI values and associated %CV for each variable separately and combined are detailed in the (S3–S5 Tables) for PBMC-A, PBMC-B, and PBMC-C, respectively. Including all variables (reagent lots, days and operators, and PBMC lots), averaged MedFI values and standard deviation (SD) of CD19 are (8824 ± 1231) with a %CV of 14 for PBMC-A, (11818 ± 1350) with a %CV of 11 for PBMC-B, and (13904 ± 3303) with a %CV of 24 for PBMC-C. Bar graph representations of the CD19 MedFI values and associated SDs are shown in Fig 2 for three different PBMC preparations.

CD19 ABC values were determined using two flow cytometry quantification approaches, CD4 reference marker approach and QuantiBrite PE calibration method and are summarized in Table 2. Using the averaged MedFI value of CD4 on Cyto-Trol cells as the reference marker, CD19 ABC values were calculated for three PBMC preparations using Eq 1 provided in ‘Materials and Methods’. Averaged CD19 ABC values are 7097 with a %CV of 12 for PBMC-A, 9505 with a %CV of 11 for PBMC-B, and 11183 with a %CV of 20 for PBMC-C (Table 2). With QuantiBrite PE calibration, averaged CD19 ABC values are 8809 with a %CV of 13 for PBMC-A, 11564 with a %CV of 11 for PBMC-B, and 13585 with a %CV of 24 for PBMC-C (Table 2).

**Table 2:** CD19 expression levels in unit of ABC and respective coefficient variations for the three PBMCs preparations.

	PBMC-A	PBMC-B	PBMC-C
CD19 ABC based on CD4 reference material*			
ABC	7097	9505	11183
%CV	12	11	20
CD19 ABC based on QuantiBrite PE beads*			
ABC	8809	11564	13585
%CV	13	11	24
***CD19 ABC based on EQ4 calibration (CyTOF)***^#^			
ABC	9296	10134	10536
%CV	6.7	12	3.7
***CD19 ABC based on ABC bead calibration (CyTOF)***^#^			
ABC	8759	9522	9886
%CV	7.5	13	4.3

*Results were obtained from 27 data points generated from 3 different PBMC lots, 3 antibody reagent lots, and 3 different experimental day and operators.

^#^CyTOF results were generated with three sample replicates carried out in three experimental dates using a single PBMC and antibody reagent lot and a single operator.

CD19 expression levels in unit of ABC along with respective coefficient variations for the three PBMCs preparations (PBMC-A, PBMC-B, and PBMC-C) using the four quantification schemes described in the method section on both flow cytometry and mass cytometry.

As an orthogonal analysis we used to CyTOF methods, EQ four calibration, and ABC bead calibration, to obtain CD19 values from a smaller set (single lots) of PBMC-A, PBMC-B, and PBMC-C. Each of these lots were included in our prior analyses. With the EQ four calibration scheme, averaged CD19 ABC values over three sample replicates are 9296 with a %CV of 6.7 for PBMC-A, 10134 with a %CV of 12 for PBMC-B, and 10536 with a %CV of 3.7 for PBMC-C (Table 2). Using ABC bead calibration, averaged CD19 ABC values over three sample replicates are 8759 with a %CV of 7.5 for PBMC-A, 9522 with a %CV of 13 for PBMC-B, and 9886 with a %CV of 4.3 for PBMC-C (Table 2).

**Fig 3:**
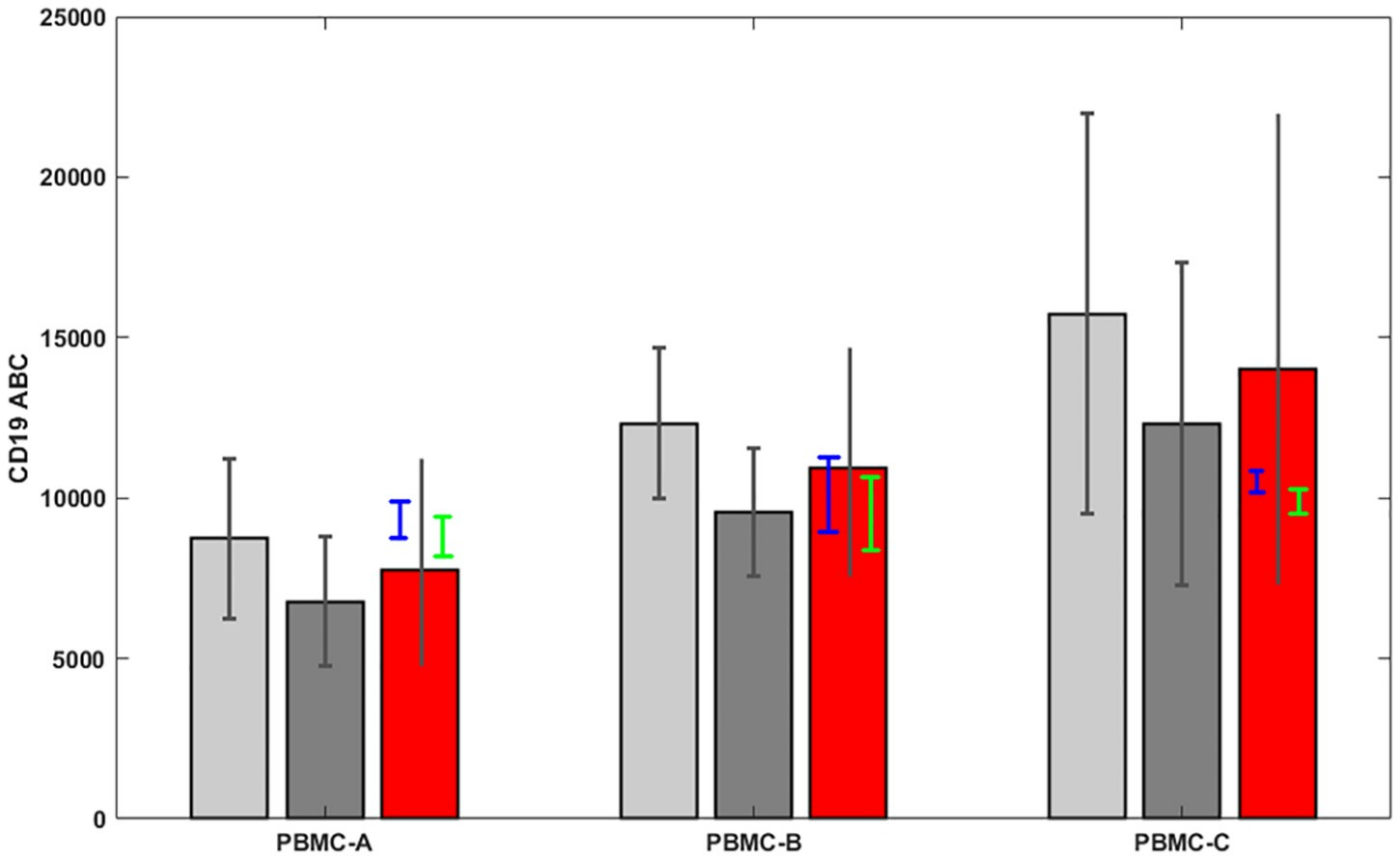
CD19 expression levels in unit of ABC and associated standard deviations at ~95% confidence level. CD19 expression levels in unit of ABC and associated standard deviations at ~95% confidence level for the three PBMCs preparations (PBMC-A, PBMC-B, and PBMC-C) using the computational method described in the method section for the two quantification approaches of flow cytometry, QuantiBrite PE calibration (light grey bar) and CD4 cell reference calibration (grey bar), and the two flow spectrometry method combined uncertainty analysis (red bar). The CyTOF quantification was conducted on a selected subset of the PBMC samples. The CD19 expression levels and associated standard deviations obtained using two CyTOF quantification methods, EQ4 calibration (blue bar) and ABC bead calibration (green bar) are also displayed within the flow cytometric results from the combined uncertainty analysis.

Fig 3 shows mean values with 2-sigma error bars (corresponding roughly to 95% confidence intervals) using 1000 realizations of the calibration parameters and 1000 realizations of Eq 4 for each set of the former, for a total of 1000000 synthetic datasets for each manufacturer-calibration pair. A combined uncertainty confidence interval is constructed by taking the union of confidence intervals for both cytometric calibration methods. The corresponding mean of combined uncertainty analysis is simply the average of the means determined by the two flow cytometry calibration methods. The mean CD19 ABC of 7700 with a range from 4700 to 11300 at a 95% confidence level was obtained for PBMC-A, 10900 with a range from 7400 to 14900 for PBMC-B, and 14000 with a range from 7200 to 22000 for PBMC-C (red bar in Fig 3).

**Fig 4:**
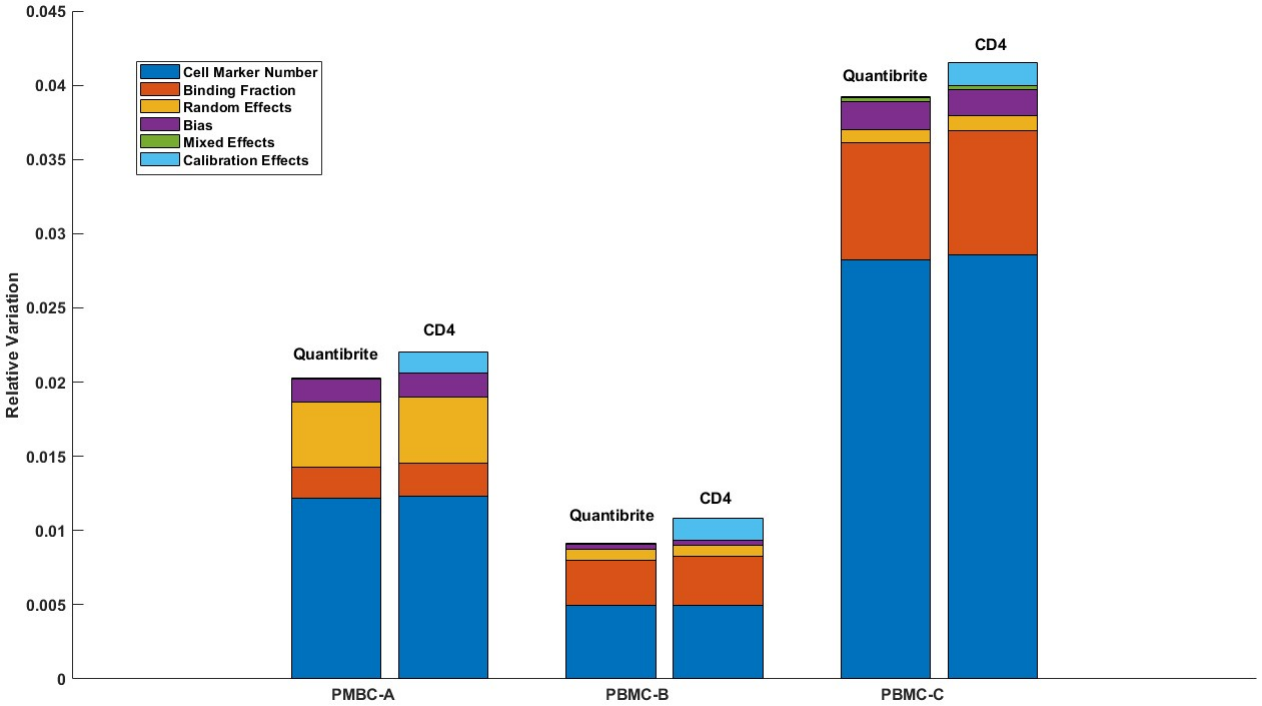
Relative variation in CD19 measurement. Relative variation (i.e. total variance divided by the mean squared) in typical CD19 measurement by PBMC manufacturer and calibration method. The relative contributions of each source of uncertainty for each PBMC preparation are indicated by the different colors.

Fig 4 shows the expected relative variations in CD19 measurement results shown in Fig 3 as a function PBMC manufacturer and calibration method. Specifically, this quantity is given by $\Delta =\varsigma^{2}/{\overset{-}{v}}^{2}$, where ς^2^ was defined previously as the total uncertainty and $\overset{-}{v}$ is the expected value of a cytometry measurement. Of note, the cell marker number is by far the largest source of variability in the measurement, accounting for between 50% and 70%. As discussed previously, it is also reassuring that the calibration method does not significantly affect the estimates for the other sources of variation, suggesting that the likelihood analysis is able to isolate their individual contributions. We also note that the use of CD4 markers for calibration leads to a small but non-trivial increase in Δ. As an aside, the relative contribution from random effects is relatively large for the PBMC-A cells. Because of the limited nature of the model, this may reflect unresolved physical phenomena (e.g. associated with antibody binding), which thereby become combined with stochastic sources of uncertainty. However, without additional information or modeling, we cannot assign a specific cause to this variability.

## Discussion

The goal of the study is to explore the characteristics of different PBMC preparations from different manufacturers with different donor numbers (single donor in PBMC-B vs. multiple donors in PBMC-A and PBMC-C) and assess the impact of uncertainties arising from all aspects including manufacturer-specific lyophilization processes, the instrumentation, antibody reagent, and sample preparation. We characterized CD19 expression and associated uncertainty in three commercial lyophilized or dried-down PBMC preparations for assessing their suitability for future use as cell reference materials for cytometric expression analysis.

Two quantification schemes of flow cytometry were applied to evaluate the consistency of CD19 expression over multiple variables in the tested cell preparations. One of the cytometric approaches employs Cyto-Trol Control Cells, a CD4 reference material with a preassigned ABC value [38,42] for translating the generated MedFI scale to the ABC scale. This CD4 ABC value was previously determined to be 40,500 using three independent methods, flow cytometry, mass cytometry, and quantitative mass spectrometry that used 4 different isotope labeled peptides as internal quantification standards [38,42]. First, we examined the consistency of CD4 expression levels in three Cyto-Trol (PBMC-A) lots using three batches of CD4 PE 1:1 antibody reagent in three different experimental days performed by three different operators (Table 1). The results show comparable MedFI values of CD4 with < 2.0% CV between different experimental days and operators for each PBMC and antibody reagent batches. Furthermore, when all four variables are included, we observed ≤ 7.0% CV in CD4 MedFI values (Table 1B). In 2018 Olga Mizrah and co-workers reported that even when using the same equipment in the same laboratory, the percentage of CV can vary between 7.0% and 33% depending on the tested marker [46]. The two-tailed, unequal variance T test identified a significant contribution from different lots of CD4 PE 1:1 conjugate to the overall variability of measured CD4 MedFI values (S2 Table). The small but non-trivial uncertainty contribution quantified by the computational analysis (Fig 4) mostly results from the manufacturing variability of this antibody reagent, which had been observed previously [37].

The second flow cytometry quantification method is based on using QuantiBrite PE bead calibration and CD19 PE 1:1 conjugate. QuantiBrite PE bead calibration enables the transformation of MedFI values of CD19 to the number of PE molecules per cell. Because of the use of CD19 PE 1:1 for cell staining, the number of PE molecules bound per cell is a close approximation of the number of antibodies bound per cell, CD19 ABC value. Since the debut of QuantiBrite PE beads in the mid 1990 [43], it is regarded as the most reliable biomarker quantification method in flow cytometry. It is important to note that this bead-based quantification scheme is only limited to PE fluorescence channel. In addition, this method relies strongly on the quality of QuantiBrite PE beads and custom-made unimolar CD19 PE conjugates. Variations in CD19 MedFI values are clearly seen between three batches of CD19 PE 1:1 conjugate for all three PBMC preparations (Fig 2 and S3–S5 Tables). Similar to the CD4 PE 1:1 quality issue discussed above, the same issue was observed and reported for other unimolar antibody reagents e.g. CD38 PE 1:1 and CD22 PE 1:1 [33,34].

In addition, CD19 ABC values were measured on selected three lots of PBMC (one per company) using two CyTOF quantification approaches (ABC bead calibration and EQ4 calibration). These quantification methods are based on the mean CyTOF intensities of the antibody stain on the cell and of the metal-encoded calibration bead(s), the independently characterized number of metal atoms per antibody molecule [47,48], and the independently characterized number of metal atoms per calibration bead [44]. The CyTOF intensity and the metal content of the bead are used to calculate the transmission coefficient for all major isotopes in the bead [49]. One method involves the use of the commercially available EQ4 beads that contain Ce, Eu, Ho, and Lu. In contrast, the second method uses a series of ABC beads with a roughly equivalent content of Ce, and with zero (ABC bead #1) and then logarithmically increased amount of La, Eu, Ho, and Lu (ABC beads #2–5). The EQ4 beads are used for a single-point calibration whereas the ABC beads allow a multi-point calibration. Test samples were run with both EQ4 and the ABC beads included. CyTOF data were not software-normalized, but instead an MS Excel sheet was used to calculate the number of antibody molecules bound per cell. Comparable CD19 ABC values were obtained between the flow cytometry and CyTOF quantification methods using a single lot of PBMC preparation. An overall averaged CD19 ABC of 9689 was obtained for all PBMCs together from the two CyTOF quantification-methods with an average of 9028 for PBMC-A, 9828 for PBMC-B, and 10211 for PBMC-C. This averaged CD19 ABC is comparable to an overall averaged CD19 ABC of 9295 for all PBMCs using the two-flow cytometry quantification-methods with an average CD19 ABC of 8014 for PBMC-A, 10399 for PBMC-B, and 9473 for PBMC-C). Several studies used similar approaches and compared the results from both flow cytometry and mass spectrometry in Immunological studies for monitoring of human cancer clinical trials [50], quantifying markers for diagnosis of Alzheimer’s [51], and measuring human hepatic transporter P-gp and OATP1B1 in hepatocytes [52].

From combined uncertainty analysis at approximately 95% confidence level, the lowest mean CD19 ABC value was obtained for PBMC-A, followed by PBMC-B and PBMC-C (red bars in Fig 3). The differences observed with the CD19 ABC values between different PBMC preparations could be related to different purification procedures from fresh, healthy whole blood samples and cell lyophilization or dried-down processes used by manufacturers. PBMC-A was trehalose sugar stabilized, unfixed lyophilized cells [53], while PBMC-B and PBMC-C were both formaldehyde-fixed mononuclear cells. After fixation, PBMC-B went through a drying process while PBMC-C was lyophilized. Scanning electron microscopy revealed that damaged and/or broken microvilli due to lyophilization process without fixation is most likely the underlying reason for low CD4 receptor density on Cyto-Trol cells (PBMC-A) respective to cryopreserved PBMC and fresh whole blood [42]. The same underlying reason could be hypothesized for the low CD19 ABC value obtained for Cyto-Trol cells (PBMC-A). Fixation prior to the lyophilization and cell drying processes could likely preserve some cell membrane structures and hence receptor epitope structures for enabling antibody binding to the CD19 receptors in PBMC-B and PBMC-C.

Currently these reference reagents are not commercially available in large quantities to serve as reference standards on a global level. These references are limited to the laboratory and need to be newly validated regularly. Users should consider the variabilities resulting from different lots of PBMC and antibody reagent when utilizing cell-based reference materials for quantification purposes and perform bridging studies to ensure harmonization between the results before switching to a new lot.

A full understanding of the sources of uncertainty, their relative contributions and areas of improvement may lead to the production of high-quality and robust reference materials for quantitative marker measurement for many application fields that are not only limited to flow cytometry. Because of the urgent need for cell reference materials for quantitative cytometric expression analysis of important clinical biomarkers, this study was designed to assess measurement variability arising from three commercially available PBMCs and external factors such as antibody reagent lots, and operators and experimental days. As candidate reference materials for CD19 expression, this uncertainty quantification is a prerequisite for directing future development of corresponding measurement standards. Though large uncertainties associated with CD19 ABC values were quantified at approximately 95% confidence level (Fig 3), this is the first time that such effort has been taken. The CD19 ABC values obtained by flow cytometry are also consistent with measurements obtained from CyTOF technology (blue and green bar in Fig 3). Importantly, the contributions from various sources of uncertainty for expression analysis beyond the designed variables specified above were quantified. The importance of analysis of the sources of uncertainty is illustrated by our finding that the primary source of variability is in the antibody reagent (Fig 4). These analysis results show needed areas for improvement, e.g. cell and reagent manufacturing, to enable the production of better cell reference materials in the future. In addition to commercially available PBMCs as potential sources of reference materials, cell lines and synthetic microbeads such as synthetic PBMCs particles will be investigated in the future using the same characterization scheme implemented in this study. The use of cultured cell lines will be conducted in order to determine the effect of additional variables in the expression analysis due to differences that might be seen in cell viability, morphology and cell sizes with different passage number.

## Conclusions

The expression levels of CD19 on B cells were quantified as well as their respective associated uncertainties for three commercial lyophilized or dried-down PBMC preparations. The work is inspired by a consensus outcome from flow cytometry workshops that call for cell reference standards with well characterized antigen expression and immunophenotyping profiles for advanced cell manufacturing and cell therapies [36]. We envision that the PBMC-based materials in this study would be useful as expression analysis reference materials for quantifying disease and immunotherapy relevant B cell markers, e.g. CD19, CD20, and CD22. Quantitative measurement of these biomarkers with high confidence is critically important for the determination of proper treatment options and regimens, e.g. switching drug and applying a second dose of the same drug, and hence, prolonging the lifespan of cancer patients with B-cell malignancies.

We are currently characterizing immunophenotyping profiles of these three different PBMC preparations that include cell subsets and T cell differentiation states. Once these PBMCs preparations are fully characterized, the users can base their application needs and tolerance of variability to choose a suitable reference PBMC material as their biological assay control for applications such as instrument set up/standardization, reagent quality control, antibody panel characterization, longitudinal studies across multi-instrument platforms and multi-centers. It’s worthy to note that these cell reference materials with assigned ABC values for CD19 and CD4 could be used for cytometric marker quantification beyond human blood T and B lymphocytes.

## Supporting information

S1 Table Respective coefficient of variations for the MedFI values of the four QuantiBrite PE peaks across the three experimental days for PBMC-A, PBMC-B and PBMC-C. (DOCX)

S2 Table CD4 MedFI values obtained using three lots of PBMC-A and three lots of CD4 antibody reagent (CD4 PE 1:1). (DOCX)

S3 Table CD19 MedFI values obtained using three lots of PBMC-A and three lots of antibody reagent (CD19 PE 1:1). (DOCX)

S4 Table CD19 MedFI values obtained using three lots of PBMC-B and three lots of antibody reagent (CD19 PE 1:1). (DOCX)

S5 Table CD19 MedFI values obtained using three lots of PBMC-C and three lots of antibody reagent (CD19 PE 1:1). (DOCX)
