# Supplementary material for: Establishing CD19 B-cell reference control materials for comparable and quantitative cytometric expression analysis
Source: PLoS One. 2021 Mar 19;16(3):e0248118. doi: 10.1371/journal.pone.0248118 (PMC7978366; doi:10.1371/journal.pone.0248118)
Supplement: S1 Table — (DOCX) [file pone.0248118.s001.docx]

# **Supplemental Table 1: Respective Coefficient of Variations for the MedFI values of the four QuantiBrite PE peaks** **across the three experimental days** **for PBMC-A, PBMC-B and PBMC-C.**

| **Table 1S: Coefficient of Variation (CV)** | | | | |
| --- | --- | --- | --- | --- |
|  | **Low** | **Med-low** | **Med-high** | **High** |
| **PBMC-A** | 0.32 | 0.41 | 0.82 | 0.24 |
| **PBMC-B** | 0.84 | 0.73 | 0.16 | 0.18 |
| **PBMC-C** | 0.74 | 0.73 | 0.50 | 0.88 |

**This is Supplemental Table 1 legend:** The coefficient of variation was < 1.0% for all intensity peaks (Low, Med-Low, Med-High and High) for PBMC-A, PBMC-B and PBMC-C
