# Supplementary material for: Establishing CD19 B-cell reference control materials for comparable and quantitative cytometric expression analysis
Source: PLoS One. 2021 Mar 19;16(3):e0248118. doi: 10.1371/journal.pone.0248118 (PMC7978366; doi:10.1371/journal.pone.0248118)
Supplement: S2 Table — (DOCX) [file pone.0248118.s002.docx]

**Supplemental Table 2:** **CD4 MedFI values obtained using three lots of PBMC-A and three lots of CD4 antibody reagent (CD4 PE 1:1)**

**This is Supplemental Table 2 legend:**  T-Tests with 2-tailed, unequal variance was carried out as shown in Table 2S assessing differences between antibody reagent lots, PBMC lots and experimental days/operators.

| **Table 2S: Two-tailed, unequal variance TTEST** | | | | | | |
| --- | --- | --- | --- | --- | --- | --- |
|  |  |  |  |  |  |  |
|  |  |  |  |  |  |  |
| **TTEST Between Reagents Lot** | | | | | | |
| R1 | 53265 | 52140 | 53634 |  | TTEST | p |
|  | 55148 | 54677 | 56294 |  | R1 vs. R2 | 4.6E-07 |
|  | 53578 | 53911 | 54916 |  | R1 vs. R3 | 8.5E-10 |
| R2 | 48977 | 48634 | 50084 |  | R2 vs. R3 | 6.0E-06 |
|  | 50706 | 50680 | 51434 |  |  |  |
|  | 48911 | 49611 | 49674 |  |  |  |
| R3 | 46674 | 46759 | 48323 |  |  |  |
|  | 46167 | 46356 | 46954 |  |  |  |
|  | 46927 | 48516 | 47027 |  |  |  |
|  |  |  |  |  |  |  |
| **TTEST Between PBMC Lots** | | | | | | |
| Lot 1 | 53265 | 52140 | 53634 |  | TTEST | p |
|  | 48977 | 48634 | 50084 |  | Lot 1 vs. Lot 2 | 0.49 |
|  | 46674 | 46759 | 48323 |  | Lot 1 vs. Lot 3 | 0.71 |
| Lot 2 | 55148 | 54677 | 56294 |  | Lot 2 vs. Lot 3 | 0.72 |
|  | 50706 | 50680 | 51434 |  |  |  |
|  | 46167 | 46356 | 46954 |  |  |  |
| Lot 3 | 53578 | 53911 | 54916 |  |  |  |
|  | 48911 | 49611 | 49674 |  |  |  |
|  | 46927 | 48516 | 47027 |  |  |  |
|  |  |  |  |  |  |  |
| **TTEST Between Days / Operator** | | | | | | |
| Day 1 | 53265 | 55148 | 53578 |  | TTEST | p |
|  | 48977 | 50706 | 48911 |  | Day 1 vs. Day 2 | 0.81 |
|  | 46674 | 46167 | 46927 |  | Day 1 vs. Day 3 | 0.58 |
| Day 2 | 52140 | 54677 | 56294 |  | Day 2 vs. Day 3 | 0.77 |
|  | 48634 | 50680 | 51434 |  |  |  |
|  | 46759 | 46356 | 46954 |  |  |  |
| Day 3 | 53634 | 56294 | 54916 |  |  |  |
|  | 50084 | 51434 | 49674 |  |  |  |
|  | 48323 | 46954 | 47027 |  |  |  |
