# Supplementary material for: Establishing CD19 B-cell reference control materials for comparable and quantitative cytometric expression analysis
Source: PLoS One. 2021 Mar 19;16(3):e0248118. doi: 10.1371/journal.pone.0248118 (PMC7978366; doi:10.1371/journal.pone.0248118)
Supplement: S3 Table — (DOCX) [file pone.0248118.s003.docx]

**Supplemental Table 3:** CD19 MedFI values obtained using three lots of PBMC-A and three lots of antibody reagent (CD19 PE 1:1).

**This is Supplemental Table 3 legend:** CD19 MedFI values obtained using three lots of PBMC-A and three lots of antibody reagent (CD19 PE 1:1) in 3 experimental days with three different operators were provided in Table 3S.A. Maximal %CV was calculated and shown in Table 3S. B for assessing uncertainty contribution from individual variable and combined variables. T-Tests with 2-tailed, unequal variance was carried out and shown in Table 3S.C assessing differences between antibody reagent lots, PBMC lots and experimental days/operators.

| **Table 3S. A** | | | | |
| --- | --- | --- | --- | --- |
| PBMC Lot | Reagent Lot | Day 1 | Day 2 | Day 3 |
| Lot 1 | R1 | 9199 | 9060 | 9900 |
|  | R2 | 8720 | 7180 | 7992 |
|  | R3 | 9070 | 9355 | 8615 |
| Lot 2 | R1 | 11275 | 9348 | 9541 |
|  | R2 | 9607 | 8036 | 8476 |
|  | R3 | 11143 | 11185 | 9751 |
| Lot 3 | R1 | 8611 | 7584 | 8507 |
|  | R2 | 6788 | 6945 | 7217 |
|  | R3 | 8845 | 9355 | 6939 |

| **Table 3S. B** | | | | |
| --- | --- | --- | --- | --- |
| CV # | PBMC Lot | Reagent Lot | Day / Operator | CV Max |
| 1 | Within | Within | Across | 12 |
| 2 | Within | Across | Within | 14 |
| 3 | Across | Within | Within | 14 |
| 4 | Within | Across | Across | 12 |
| 5 | Across | Within | Across | 13 |
| 6 | Across | Across | Within | 15 |
| 7 | Across | Across | Across | 14 |

| **Table 3S.C: Two-tailed, unequal variance TTEST** | | | | | | |
| --- | --- | --- | --- | --- | --- | --- |
|  |  |  |  |  |  |  |
|  |  |  |  |  |  |  |
| **TTEST Between Reagents Lot** | | | | | | |
| R1 | 9199 | 9060 | 9900 |  | TTEST | p |
|  | 11275 | 9348 | 9541 |  | R1 vs. R2 | 0.01 |
|  | 8611 | 7584 | 8507 |  | R1 vs. R3 | 0.81 |
| R2 | 8720 | 7180 | 7992 |  | R2 vs. R3 | 0.01 |
|  | 9607 | 8036 | 8476 |  |  |  |
|  | 6788 | 6945 | 7217 |  |  |  |
| R3 | 9070 | 9355 | 8615 |  |  |  |
|  | 11143 | 11185 | 9751 |  |  |  |
|  | 8845 | 9355 | 6939 |  |  |  |
|  |  |  |  |  |  |  |
| **TTEST Between PBMC Lots** | | | | | | |
| Lot 1 | 9199 | 9060 | 9900 |  | TTEST | p |
|  | 8720 | 7180 | 7992 |  | Lot 1 vs. Lot 2 | 0.05 |
|  | 9070 | 9355 | 8615 |  | Lot 1 vs. Lot 3 | 0.04 |
| Lot 2 | 11275 | 9348 | 9541 |  | Lot 2 vs. Lot 3 | 1.5E-03 |
|  | 9607 | 8036 | 8476 |  |  |  |
|  | 11143 | 11185 | 9751 |  |  |  |
| Lot 3 | 8611 | 7584 | 8507 |  |  |  |
|  | 6788 | 6945 | 7217 |  |  |  |
|  | 8845 | 9355 | 6939 |  |  |  |
|  |  |  |  |  |  |  |
| **TTEST Between Days / Operator** | | | | | | |
| Day 1 | 9199 | 11275 | 8611 |  | TTEST | p |
|  | 8720 | 9607 | 6788 |  | Day 1 vs. Day 2 | 0.81 |
|  | 9070 | 11143 | 8845 |  | Day 1 vs. Day 3 | 0.24 |
| Day 2 | 9060 | 9348 | 9541 |  | Day 2 vs. Day 3 | 0.30 |
|  | 7180 | 8036 | 8476 |  |  |  |
|  | 9355 | 11185 | 9751 |  |  |  |
| Day 3 | 9900 | 9541 | 8507 |  |  |  |
|  | 7992 | 8476 | 7217 |  |  |  |
|  | 8615 | 9751 | 6939 |  |  |  |
