# Supplementary material for: Establishing CD19 B-cell reference control materials for comparable and quantitative cytometric expression analysis
Source: PLoS One. 2021 Mar 19;16(3):e0248118. doi: 10.1371/journal.pone.0248118 (PMC7978366; doi:10.1371/journal.pone.0248118)
Supplement: S4 Table — (DOCX) [file pone.0248118.s004.docx]

**Supplemental Table 4:** CD19 MedFI values obtained using three lots of PBMC-B and three lots of antibody reagent (CD19 PE 1:1)

**This is Supplemental Table 4 legend:**  CD19 MedFI values obtained using three lots of PBMC-B and three lots of antibody reagent (CD19 PE 1:1) in 3 experimental days with three different operators were provided in Table 4S.A. Maximal %CV was calculated and shown in Table 4S.B for assessing uncertainty contribution from individual variable and combined variables. T-Tests with 2-tailed, unequal variance was carried out and shown in Table 4S.C assessing differences between antibody reagent lots, PBMC lots and experimental days/operators.

| **Table 4S. A** | | | | |
| --- | --- | --- | --- | --- |
| PBMC Lot | Reagent Lot | Day 1 | Day 2 | Day 3 |
| Lot 1 | R1 | 12764 | 12371 | 12278 |
|  | R2 | 11570 | 10897 | 11072 |
|  | R3 | 13269 | 13848 | 14394 |
| Lot 2 | R1 | 10981 | 10210 | 10877 |
|  | R2 | 9847 | 9151 | 9940 |
|  | R3 | 11517 | 12602 | 12774 |
| Lot 3 | R1 | 11892 | 11600 | 12156 |
|  | R2 | 10996 | 10331 | 11117 |
|  | R3 | 12907 | 13644 | 14074 |

| **Table 4S. B** | | | | |
| --- | --- | --- | --- | --- |
| CV # | PBMC Lot | Reagent Lot | Day / Operator | CV Max |
| 1 | Within | Within | Across | 4.5 |
| 2 | Within | Across | Within | 14 |
| 3 | Across | Within | Within | 7.8 |
| 4 | Within | Across | Across | 11 |
| 5 | Across | Within | Across | 6.9 |
| 6 | Across | Across | Within | 13 |
| 7 | Across | Across | Across | 11 |

| **Table 4S.C: Two-tailed, unequal variance TTEST** | | | | | | |
| --- | --- | --- | --- | --- | --- | --- |
|  |  |  |  |  |  |  |
|  |  |  |  |  |  |  |
| **TTEST Between Reagents Lot** | | | | | | |
| R1 | 12764 | 12371 | 12278 |  | TTEST | p |
|  | 10981 | 10210 | 10877 |  | R1 vs. R2 | 0.01 |
|  | 11892 | 11600 | 12156 |  | R1 vs. R3 | 1.6E-03 |
| R2 | 11570 | 10897 | 11072 |  | R2 vs. R3 | 4.4E-06 |
|  | 9847 | 9151 | 9940 |  |  |  |
|  | 10996 | 10331 | 11117 |  |  |  |
| R3 | 13269 | 13848 | 14394 |  |  |  |
|  | 11517 | 12602 | 12774 |  |  |  |
|  | 12907 | 13644 | 14074 |  |  |  |
|  |  |  |  |  |  |  |
| **TTEST Between PBMC Lots** | | | | | | |
| Lot 1 | 12764 | 12371 | 12278 |  | TTEST | p |
|  | 11570 | 10897 | 11072 |  | Lot 1 vs. Lot 2 | 0.01 |
|  | 13269 | 13848 | 14394 |  | Lot 1 vs. Lot 3 | 0.48 |
| Lot 2 | 10981 | 10210 | 10877 |  | Lot 2 vs. Lot 3 | 0.06 |
|  | 9847 | 9151 | 9940 |  |  |  |
|  | 11517 | 12602 | 12774 |  |  |  |
| Lot 3 | 11892 | 11600 | 12156 |  |  |  |
|  | 10996 | 10331 | 11117 |  |  |  |
|  | 12907 | 13644 | 14074 |  |  |  |
|  |  |  |  |  |  |  |
| **TTEST Between Days / Operator** | | | | | | |
| Day 1 | 12764 | 10981 | 11892 |  | TTEST | p |
|  | 11570 | 9847 | 10996 |  | Day 1 vs. Day 2 | 0.60 |
|  | 13269 | 11517 | 12907 |  | Day 1 vs. Day 3 | 0.60 |
| Day 2 | 12371 | 10210 | 10877 |  | Day 2 vs. Day 3 | 0.37 |
|  | 10897 | 9151 | 9940 |  |  |  |
|  | 13848 | 12602 | 12774 |  |  |  |
| Day 3 | 12278 | 10877 | 12156 |  |  |  |
|  | 11072 | 9940 | 11117 |  |  |  |
|  | 14394 | 12774 | 14074 |  |  |  |
