# Supplementary material for: Establishing CD19 B-cell reference control materials for comparable and quantitative cytometric expression analysis
Source: PLoS One. 2021 Mar 19;16(3):e0248118. doi: 10.1371/journal.pone.0248118 (PMC7978366; doi:10.1371/journal.pone.0248118)
Supplement: S5 Table — (DOCX) [file pone.0248118.s005.docx]

**Supplemental Table 5:** CD19 MedFI values obtained using three lots of PBMC-C and three lots of antibody reagent (CD19 PE 1:1)

**This is Supplemental Table 5 legend:** CD19 MedFI values obtained using three lots of PBMC-C and three lots of antibody reagent (CD19 PE 1:1) in 3 experimental days with three different operators were provided in Table 5S.A. Maximal %CV was calculated and shown in Table 5S.B for assessing uncertainty contribution from individual variable and combined variables. T-Tests with 2-tailed, unequal variance was carried out and shown in Table 5S.C assessing differences between antibody reagent lots, PBMC lots and experimental days/operators.

| **Table 5S. A** | | | | |
| --- | --- | --- | --- | --- |
| PBMC Lot | Reagent Lot | Day 1 | Day 2 | Day 3 |
| Lot 1 | R1 | 13592 | 13199 | 13228 |
|  | R2 | 11445 | 11013 | 11479 |
|  | R3 | 17497 | 15658 | 18479 |
| Lot 2 | R1 | 10913 | 10118 | 10997 |
|  | R2 | 9181 | 8523 | 9912 |
|  | R3 | 14553 | 13132 | 16272 |
| Lot 3 | R1 | 15368 | 14132 | 15193 |
|  | R2 | 13679 | 12791 | 14051 |
|  | R3 | 20707 | 19313 | 20981 |

| **Table 5S. B** | | | | |
| --- | --- | --- | --- | --- |
| CV # | PBMC Lot | Reagent Lot | Day / Operator | CV Max |
| 1 | Within | Within | Across | 8.8 |
| 2 | Within | Across | Within | 22 |
| 3 | Across | Within | Within | 16 |
| 4 | Within | Across | Across | 21 |
| 5 | Across | Within | Across | 16 |
| 6 | Across | Across | Within | 24 |
| 7 | Across | Across | Across | 24 |

| **Table 5S.C: Two-tailed, unequal variance TTEST** | | | | | | |
| --- | --- | --- | --- | --- | --- | --- |
|  |  |  |  |  |  |  |
|  |  |  |  |  |  |  |
| **TTEST Between Reagents Lot** | | | | | | |
| R1 | 13592 | 13199 | 13228 |  | TTEST | p |
|  | 10913 | 10118 | 10997 |  | R1 vs. R2 | 0.09 |
|  | 15368 | 14132 | 15193 |  | R1 vs. R3 | 1.2E-03 |
| R2 | 11445 | 11013 | 11479 |  | R2 vs. R3 | 7.7E-05 |
|  | 9181 | 8523 | 9912 |  |  |  |
|  | 13679 | 12791 | 14051 |  |  |  |
| R3 | 17497 | 15658 | 18479 |  |  |  |
|  | 14553 | 13132 | 16272 |  |  |  |
|  | 20707 | 19313 | 20981 |  |  |  |
|  |  |  |  |  |  |  |
| **TTEST Between PBMC Lots** | | | | | | |
| Lot 1 | 13592 | 13199 | 13228 |  | TTEST | p |
|  | 11445 | 11013 | 11479 |  | Lot 1 vs. Lot 2 | 0.07 |
|  | 17497 | 15658 | 18479 |  | Lot 1 vs. Lot 3 | 0.12 |
| Lot 2 | 10913 | 10118 | 10997 |  | Lot 2 vs. Lot 3 | 3.4E-03 |
|  | 9181 | 8523 | 9912 |  |  |  |
|  | 14553 | 13132 | 16272 |  |  |  |
| Lot 3 | 15368 | 14132 | 15193 |  |  |  |
|  | 13679 | 12791 | 14051 |  |  |  |
|  | 20707 | 19313 | 20981 |  |  |  |
|  |  |  |  |  |  |  |
| **TTEST Between Days / Operator** | | | | | | |
| Day 1 | 13592 | 10913 | 15368 |  | TTEST | p |
|  | 11445 | 9181 | 13679 |  | Day 1 vs. Day 2 | 0.19 |
|  | 17497 | 14553 | 20707 |  | Day 1 vs. Day 3 | 0.81 |
| Day 2 | 13199 | 10118 | 10997 |  | Day 2 vs. Day 3 | 0.13 |
|  | 11013 | 8523 | 9912 |  |  |  |
|  | 15658 | 13132 | 16272 |  |  |  |
| Day 3 | 13228 | 10997 | 15193 |  |  |  |
|  | 11479 | 9912 | 14051 |  |  |  |
|  | 18479 | 16272 | 20981 |  |  |  |
